# Does Thoracic Duct Ligation at the Time of Esophagectomy Impact Long-Term Survival? An Individual Patient Data Meta-Analysis

**DOI:** 10.3390/jcm13102849

**Published:** 2024-05-12

**Authors:** Alberto Aiolfi, Davide Bona, Matteo Calì, Michele Manara, Emanuele Rausa, Gianluca Bonitta, Moustafa Elshafei, Sheraz R. Markar, Luigi Bonavina

**Affiliations:** 1 IRCCS Ospedale Galeazzi—Sant’Ambrogio, Division of General Surgery, Department of Biomedical Science for Health, University of Milan, 20157 Milan, Italy; davide.bona@unimi.it (D.B.);; 2 Department of Bariatric and Metabolic Medicine, Clinic Northwest, 60488 Frankfurt, Germany; elshafei.moustafa@gmail.com; 3 Nuffield Department of Surgical Sciences, University of Oxford, Oxford OX1 2JD, UK; sheraz.markar@nds.ox.ac.uk; 4 IRCCS Policlinico San Donato, Division of General and Foregut Surgery, Department of Biomedical Sciences for Health, University of Milan, 20097 Milan, Italy; luigi.bonavina@unimi.it

**Keywords:** esophagectomy, esophageal cancer, thoracic duct ligation, overall survival, chylothorax

## Abstract

**Background**: Thoracic duct ligation (TDL) during esophagectomy has been proposed to reduce the risk of postoperative chylothorax. Because of its role in immunoregulation, some authors argued that it had an unfavorable TDL effect on survival. The aim of this study was to analyze the effect of TDL on overall survival (OS). **Methods**: PubMed, MEDLINE, Scopus, and Web of Science were searched through December 2023. The primary outcome was 5-year OS. The restricted mean survival time difference (RMSTD), hazard ratios (HRs), and 95% confidence intervals (CI) were used as pooled effect size measures. The GRADE methodology was used to summarize the certainty of the evidence. **Results**: Five studies (3291 patients) were included. TDL was reported in 54% patients. The patients’ age ranged from 49 to 69, 76% were males, and BMI ranged from 18 to 26. At the 5-year follow-up, the combined effect from the multivariate meta-analysis is -3.5 months (95% CI −6.1, −0.8) indicating that patients undergoing TDL lived 3.5 months less compared to those without TDL. TDL was associated with a significantly higher hazard for mortality at 12 months (HR 1.54, 95% CI 1.38–1.73), 24 months (HR 1.21, 95% CI 1.12–1.35), and 28 months (HR 1.14, 95% CI 1.02–1.28). TDL and noTDL seem comparable in terms of the postoperative risk for chylothorax (RR = 0.66; *p* = 0.35). **Conclusions**: In this study, concurrent TDL was associated with reduced 5-year OS after esophagectomy. This may suggest the need of a rigorous follow-up within the first two years of follow-up.

## 1. Introduction

The thoracic duct plays a fundamental role in maintaining body homeostasis since it drains filtered chyle through the venous system [1]. Chyle contains emulsified fats, proteins, sugars, lymphocytes, and immunoglobulins, thus contributing to the maintenance of fluid balance, fat absorption, and immune function [2,3,4]. Iatrogenic thoracic duct injury may occur during esophagectomy, thus leading to postoperative chylothorax reported in up to 4% of patients [5,6,7]. Thoracic duct ligation (TDL) during esophagectomy has been proposed for a long time in an attempt to reduce the risk of postoperative chylothorax [8].

Because of its central role in immunoregulation, some authors argued that there was an unfavorable effect of TDL on the 5-year overall survival (OS) while two recent meta-analyses reported no significant differences [9,10]. However, selection bias, heterogeneity related to inclusions of studies reporting thoracic duction resection, and the exclusive utilization of HRs constitute limitations. Further, the unique interpretation of HRs in previous meta-analyses may be misleading because these are time-dependent values and may change over time. For these reasons, we performed an updated individual patient data (IPD) meta-analysis with a restricted mean survival time estimation difference (RMSTD) with the intent to assess the effect of TDL on 5-year OS.

## 2. Materials and Methods

A systematic review was reported following the Preferred Reporting Items for Systematic Reviews and Meta-Analyses checklist guideline (PRISMA 2020) [11]. Ethical approval was not required. Scopus, MEDLINE, Web of Science, ClinicalTrials.gov, Cochrane Central Library, and Google Scholar were used [12]. The first search was run in January 2024, repeated in February 2024, and finally updated on 20 February 2024. A combination of the following Medical Subject Headings (MeSH) terms was used: “Esophageal Cancer”, “Esophageal Neoplasm”, “Esophageal Carcinoma”, “Esophagectomy”, “Esophageal resection”, “survival”, “overall survival”, “thoracic duct ligation”, “thoracic duct obliteration”, and “chylothorax”. The complete search strategy is labelled in the supplementary file. All titles were screened, suitable abstracts were obtained, and the reference lists of each article were appraised independently by three authors (AA, MC, and MM). Study was registered with PROSPERO (CRD42024505578).

### 2.1. Eligibility Criteria

The inclusion criteria comprised the following: (1) observational and randomized controlled studies that presented long-term survival information or Kaplan–Meier survival curves comparing thoracic duct ligation (TDL) with noTDL in the context of curatively aimed esophagectomy; (2) in instances where multiple articles were published by the same institution, study group, or based on the same dataset, the articles with the longest follow-up duration or the largest sample size were selected; and (3) for duplicate studies, the most recent and comprehensive reports were chosen. The exclusion criteria were: (1) studies not written in English; (2) studies lacking a comparative analysis between TDL and noTDL; (3) studies that did not report the predefined primary outcome (OS); (4) studies focusing on outcomes of thoracic duct resection; and (5) studies with fewer than 10 patients in each study arm.

### 2.2. Data Extraction

The following data points were gathered: authors, publication year, country, study design, number of patients, gender, age, body mass index (BMI), American Society of Anesthesiologists (ASA) physical status, comorbidities, tumor characteristics, tumor location, surgical approach, postoperative outcomes, pathological outcomes, necessity for postoperative adjuvant treatment, duration of follow-up, and overall survival (OS). Two authors (A.A. and G.B.) independently collected all data and reconciled any disparities during the evaluation process. Subsequently, a third author (LB) reviewed the database and resolved any inconsistencies.

### 2.3. Outcome of Interest and Definition

The primary outcome assessed was overall survival (OS), which was described as the duration from the surgery date to the most recent follow-up and demise. Secondary outcomes included incidents of chylothorax and anastomotic leak. Information regarding OS was derived from Kaplan–Meier survival curves or obtained from reported hazard ratios (HRs). Esophageal cancer was defined as any primary histopathologically verified neoplasm situated in the cervical, thoracic, and distal esophagus (Siewert I–II).

### 2.4. Quality Assessment and Assessment of Certainty of Evidence

Two authors (AA, and GB) independently evaluated the methodological quality of the included papers utilizing the ROBINS-I tool [13]. Factors such as confounding, selection, classification, intervention, missing data, outcome measurement, and reporting bias were taken into consideration, with each domain being categorized as “low”, “moderate”, “serious”, or “critical”. The assessment for confounding bias in each study fell into the risk categories of low, moderate, serious, and critical. The quality of the body of evidence across studies was appraised using the Grading of Recommendations, Assessment, Development, and Evaluation (GRADE) tool [14]. GRADE evidence profiles were created for each comparison and outcome using GRADEpro GDT software (https://www.gradepro.org; accessed on 10 March 2024). The certainty of evidence was determined by factors such as the risk of bias across studies, incoherence, indirectness, imprecision, publication bias, and other relevant parameters [15].

### 2.5. Statistical Analysis

The systematic review findings were qualitatively summarized and transformed into a Frequentist meta-analysis of the restricted mean survival time difference (RMSTD) [16,17,18]. Individual patient time-to-event data (IPD) were reconstructed from Kaplan–Meier curves [19] by digitizing the curves through the Get Data Graph Digitizer software version 2.26 (https://getdata-graph-digitizer.software.informer.com/; accessed on 20 February 2024). The calculation of the pooled RMSTD was conducted using a random-effect multivariate meta-analysis that leveraged strength across time-points with within-trial covariance. Furthermore, a flexible hazard-based regression model was developed using IPD, incorporating a normally distributed random intercept. In modeling the baseline hazard within periocular, an exponential of a B-spline of degree 3 without interior knots was employed, with model selection based on the Akaike Information Criterion (AIC). The time-varying effects of surgical treatment were represented as interaction terms between the surgical intervention and the baseline hazard, evaluated through likelihood ratio tests. The hazard functions plot was generated using marginal prediction [20]. Statistical significance was determined by two-sided *p*-values less than 0.05, with confidence intervals computed at 95%. The statistical analysis was performed using R software application (version 3.2.2; R Foundation, Vienna, Austria) [21].

## 3. Results

### 3.1. Systematic Review

**Figure 1:**
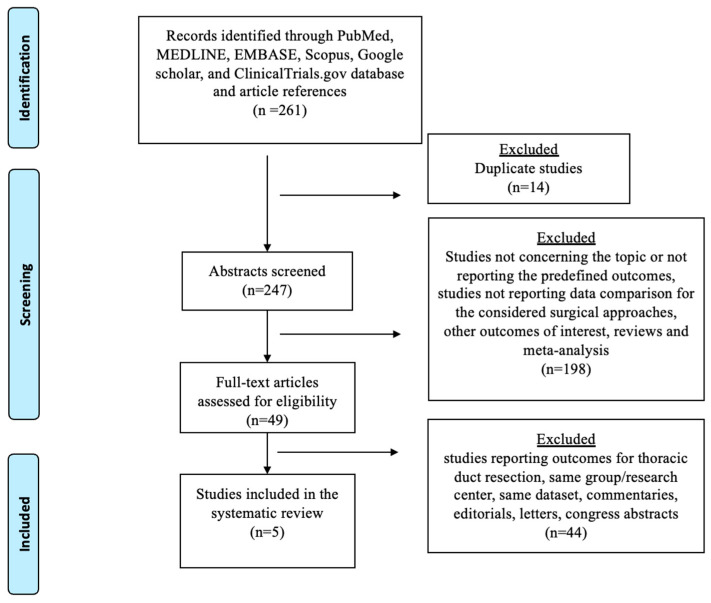
The Preferred Reporting Items for Systematic Reviews checklist (PRISMA) diagram.

The selection process flowchart is shown in Figure 1. Overall, 247 publications were screened after duplicate removal, and 49 were identified for the full-text review. After evaluation, five observational papers met the inclusion and exclusion criteria and were included in the quantitative analysis. The quality of the included studies is listed in Supplementary Table S1.

Overall, 3291 patients undergoing esophagectomy for cancer were incorporated for quantitative synthesis (Table 1).

TDL was reported in 1778 (54%) patients. The patients’ age ranged from 49 to 69, the majority were males (76.1%), and the preoperative BMI ranged from 18 to 26. None of the included studies reported data on the ASA score. Squamous cell carcinoma (89.5%) and adenocarcinoma (8.2%) were the most frequently reported tumor histology. Tumor location was reported in all studies and distributed in the upper (16.9%), middle (59.8%), lower thoracic (17.6%) esophagus, and esophagogastric junction (5.7%). Neoadjuvant chemoradiation treatment was reported in 7.2% of patients while adjuvant therapy was completed in 17.4% of patients with different protocols and regimens. Multiple surgical techniques of esophagectomy were reported (Ivor–Lewis, McKeown, and Sweet) and included open, hybrid, and minimally invasive approaches. Two-field and three-field lymphadenectomy were described, whereas anastomotic techniques and location were heterogeneous among the included studies, mainly depending on operating surgeon preferences. The pathological tumor stage was reported according to the seventh and eighth edition of the American Joint Committee on Cancer. The techniques for TDL and definitions of chylothorax according to the included studies are summarized in Supplementary Tables S2 and S3.

### 3.2. Meta-Analysis

#### 3.2.1. Overall Survival (OS)

**Figure 2:**
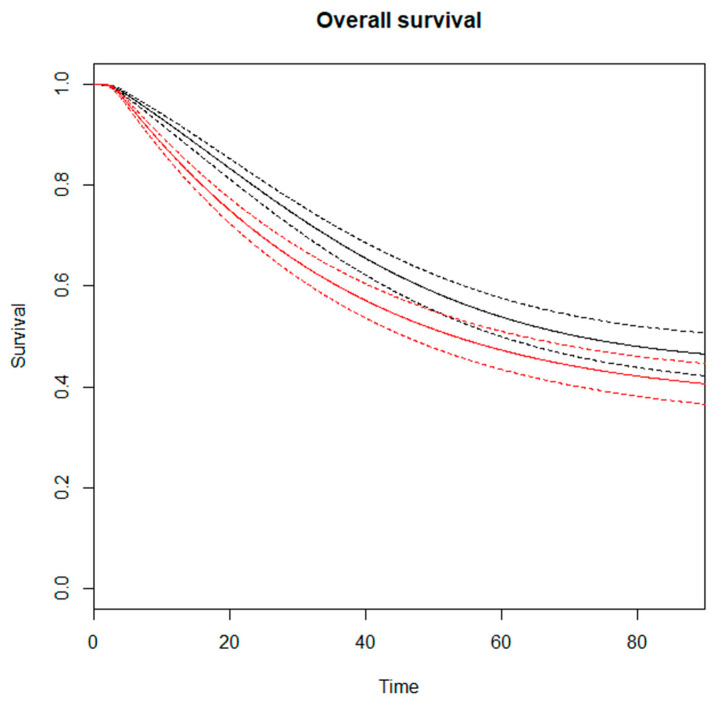
Estimated pooled OS (*Y*-axis) for NTDL (black line) and TDL (red line). Time (*X*-axis) is expressed in months. Continuous lines indicate survival curves with 95% confidence intervals (dotted lines).

**Figure 3:**
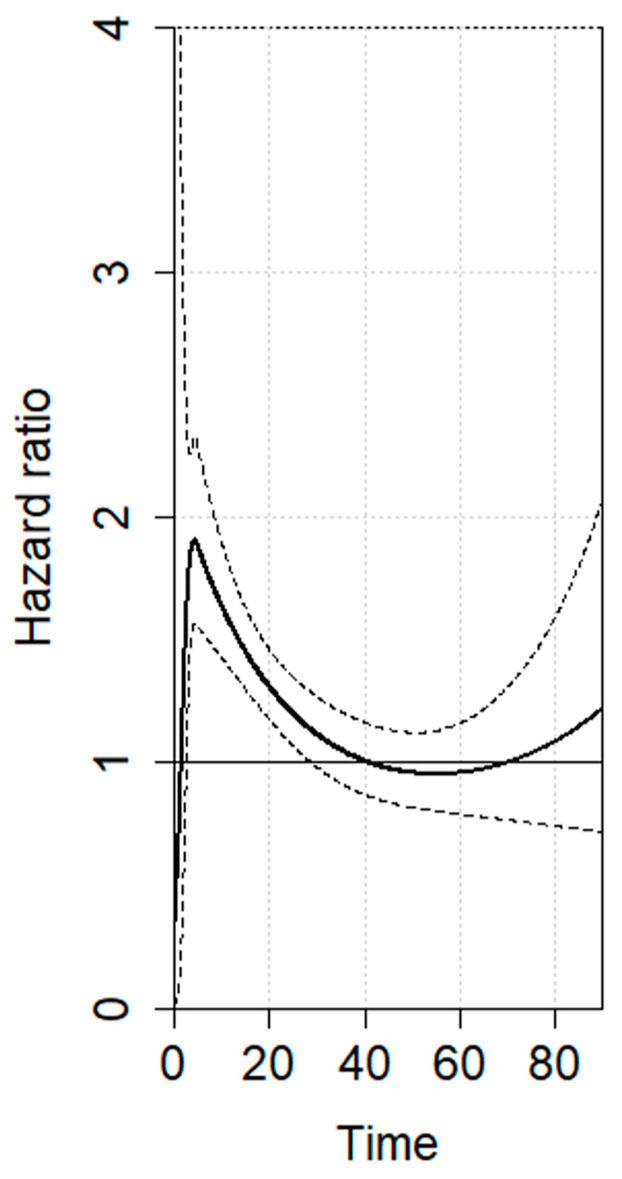
TDL vs. noTDL overtime OS hazard ratio (HR) variations (*Y*-axis). Continued tracts represent the estimated pooled hazards while dotted tracts represent the 95% Confidence Interval (95% CI). Time (*X*-axis) is expressed in months. Continuous lines indicate survival curves with 95% confidence intervals (dotted lines).

The RMSTD clinical appraisal was estimated from five studies with a 5-year minimum follow-up [14,18,19,20,22,23]. The RMSTD and OS time horizons were performed for the comparison between noTDL vs. TDL (Table 2). At τ2 = 24-month follow-up, the combined effect from the RMSTD estimate is −1.1 months (95% CI −1.8, −0.4). At τ3 = 36-month, the combined effect is −2.1 months (95% CI −3.3, −0.7). At τ4 = 48-month, the combined effect is −2.8 months (95% CI −4.8, −0.8). At τ5 = 60-month, the combined effect from the multivariate meta-analysis is −3.5 months (95% CI −6.1, −0.8), indicating that the over-5-year-follow-up patients that underwent TDL lived 3.5 months less on average compared with noTDL patients. The estimated pooled OS for TDL and noTDL is represented in Figure 2. Considering the non-proportional hazard model (*p* < 0.001), the time-varying hazard ratios for TDL vs. noTDL are depicted in Figure 3. Specifically, TDL is associated with a significantly estimated higher hazard for mortality at 6 months (HR 1.83, 95% CI 1.53–2.21), 12 months (HR 1.54, 95% CI 1.38–1.73), 24 months (HR 1.21, 95% CI 1.12–1.35), and 28 months (HR 1.14, 95% CI 1.02–1.28) compared to noTDL (Table 3).

#### 3.2.2. Secondary Outcomes

**Figure 4:**
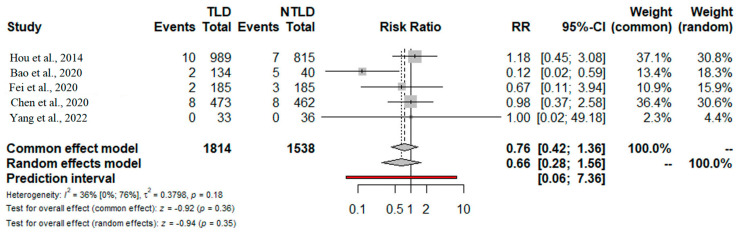
Forest plot for postoperative chylothorax. TDL: thoracic duct ligation; noTDL: no thoracic duct ligation. RR: risk ratio; 95% Confidence Interval (95% CI) [22,23,24,25,26].

The incidence of chylothorax was reported in all included studies (3291 patients) and was comparable for TDL vs. noTDL (1.2% vs. 1.5%). No significant differences were found for the comparison between TDL vs. noTDL (RR = 0.66; 95% CI 0.28–1.56; *p* = 0.35) (Figure 4). The prediction lower and upper limits were 0.06 and 7.36, respectively. The heterogeneity was low (I^2^ = 21%, 95% CI 0.0–76%; *p* = 0.18) and τ2 = 0.01. The sensitivity analysis confirmed the strength of these results regarding the point estimate, relative confidence intervals, and heterogeneity. Due to AL being reported in only two studies, a comprehensive quantitative analysis was not viable. By employing the GRADE tool, the level of evidence for overall survival (OS) was deemed moderate, while for chylothorax, it was considered high (Supplementary Table S4).

## 4. Discussion

This IPD meta-analysis shows that TDL has a moderately negative impact on OS. At the 60-month follow-up, the OS of patients who underwent TDL was reduced by 3.5 months on average, whereas no remarkable difference was found between the two patients’ groups in terms of postoperative chylothorax.

Esophagectomy is the mainstay of treatment for resectable esophageal cancer [27,28]. The surgical procedure is complex and associated with significant postoperative morbidity and mortality [29,30,31]. Postoperative chylothorax occurs in up to 4% of patients and is determined by iatrogenic thoracic duct injury during surgical maneuvers [5,6,7]. Chylous leakage results in fluid loss, hypovolemia, electrolyte imbalance, nutritional and metabolic depletion, impaired immunological response, and the increased risk of postoperative death. Prophylactic TDL, first performed in 1948 [32], has been proposed during esophagectomy in order to theoretically reduce the risk of postoperative chylothorax or increase the number of harvested lymph nodes [8,33,34,35]. The selective thoracic duct ligature or blind mass ligation of the lympho-fatty tissue located between the aorta and the azygos vein at the level of the eighth–ninth thoracic vertebra is the classical technique for TDL [36]. It should be noted that attempts to identify and ligate the duct, selectively or en bloc with the azygos vein, may lead to inadvertent injury and the occurrence of iatrogenic chylothorax [37]. With new developments in thoracoscopic and robotic-assisted surgery and a better understanding of esophageal anatomy, it has become clear that the quality and reproducibility of esophagectomy can be improved [38]. ICG guidance has the potential to precisely identify the course of the duct in the chest and to avoid possible chyle leakage due to inadvertent injury [39,40,41]. All studies included in the present systematic review reported blind thoracic duct ligation using different anatomical landmarks (Supplementary Table S2). The impact of prophylactic TDL on postoperative chylothorax is a matter of debate. Dougenis et al., in their 1992 retrospective analysis, indicated a possible benefit for TDL in terms of postoperative chylothorax incidence reduction [42]. Crucitti et al., in their 2016 systematic review, concluded that prophylactic TDL may be associated with significantly reduced odds for postoperative chylothorax (OR = 0.47; 95% CI 0.27–0.80) [8]. Similarly, Guo et al. [34], Cagol et al. [43], and Lai et al. [44] identified a trend toward a reduced risk of postoperative chylothorax for TDL. In contrast, Fu et al. [45], in their series of 389 patients, reported no significant differences between TDL vs. noTDL (1.2% vs. 0.5%) in terms of postoperative chylothorax. Further, a recent meta-analysis by Liu et al. [10], re-elaborating some missing data by Crucitti et al. [8], found no significant differences in terms of postoperative chylothorax (OR = 0.73; 95% CI 0.50–1.07). Our meta-analysis supports the data by Fu et al. [45] and Lei et al. [10] reporting a similar postoperative risk for TDL vs. noTDL (RR = 0.66; 95% CI 0.28–1.56). The heterogeneity was low (I^2^ = 21%) while the sensitivity analysis seems to confirm the robustness of these data. Therefore, it is likely that routine prophylactic TDL during esophagectomy is unnecessary.

The oncological impact of TDL is a more complex issue and the evidence from the literature evidence is puzzling. In our study, we observed that the 5-year life-expectancy of patients that underwent prophylactic TDL was reduced by 3.5 months compared to noTDL patients. This is in line with the findings of Chen et al. [25], who, in their propensity-score-adjusted analysis, concluded that TDL was associated with a worse 5-year cumulative survival compared to patients that did not undergo TDL (48.6% vs. 61.6%; *p* < 0.001). In the regression analysis, TDL was an independent predictor of poor prognosis (HR = 1.56; 95% CI 1.26–1.93; *p* < 0.001). Similarly, Hou et al. [22] reported reduced 5-year (46.1% vs. 43.3%) and 10-year (35.1% vs. 30.9%) OS rates in patients that underwent TDL. The multivariate analysis adjusted for clinically relevant confounders found that TDL was independently associated with poor OS (HR = 1.25; 95% CI 1.08–1.46). In contrast, Bao et al. [23] and Fei and colleagues [24] did not report any significant effect of TDL on patient prognosis. Our results may be plausible due to the central homeostatic role of the thoracic duct in the physiology of immunoregulation. Specifically, the thoracic duct carries emulsified fats, together with the lymph to maintain the drainage of a normal immune system. T lymphocytes and other immune components (cytokines) enter the lymphatic tissue and lymphoid organ via postcapillary venues, and then through the thoracic duct, and into superior vena cava [1,4]. Indeed, TDL may modify the pattern of lymph circulation, possibly altering the physiological immune function barrier against circulating cancer cells. Further, it has been suggested that TDL may decrease the concentration of circulating CD4+ lymphocyte, IL-1 beta, and IL-10 in peripheral blood samples [46,47,48]. All these mutual modifications may trigger a significant lymphocyte-mediate immune dysfunction against circulating cancer cell and lymphatic micro metastases with an increased risk of locoregional lymph nodes relapse and early distal organ metastases [47,48]. Hence, all these reasons might be directly involved with the observed TDL-related OS reduction perceived in the RMSTD analysis.

Because of the lack of proportionality (*p* < 0.001), we performed a time-dependent HR analysis. It is well-known that HRs change over time and are useful to describe the magnitude and direction of survival outcomes [49]. Notably, two previous meta-analyses assessed HR by reporting a single 5-year follow-up calculation that was presumed constant over the entire duration of the study [9,10]. However, the single interpretation of HRs may be misleading because of the time-dependent modifications. As expected, in our analysis, we noticed some HR variations during follow-up (Figure 3). Interestingly, when looking at the shape of the time-dependent HR curve, there is an initial peak (HR = 1.9) presumably associated with the increased postoperative mortality. After this initial peak, the curve takes a downward trend with a gradual HR reduction which, however, remains significant up to 28 months (Figure 3). At this time-point, the HR lower 95% CI bundle encompasses the null hypothesis (HR = 1), thus becoming not significant. This dynamic effect may be attributed to a temporary impairment of the immunological system and impairment of lipo-protein absorption induced by TDL which may last up to 2 years after resection. Therefore, these patients might benefit from a more intensive and patient-tailored surveillance strategy to detect possible early asymptomatic recurrences [50,51,52].

With the aim to minimize heterogeneity, we included in our meta-analysis only studies reporting survival outcomes after TDL. The formal thoracic duct resection (TDR) has been recommended to achieve a complete lymphadenectomy of the posterior mediastinal compartment including lymph node stations no. 106recL and no. 112 as defined by Japanese guidelines [53,54,55,56,57]. The potential advantages of TDR are a higher total number of retrieved lymph nodes and a reduced risk of nodal and hematogenous recurrence after radical esophagectomy [58,59,60,61]. However, TDR seems associated with an increased risk of short-term adverse outcomes such as chylothorax, left recurrent nerve palsy, pulmonary complications, and hemodynamic instability that may be somewhat related to the concomitant azygos vein ligation/resection [60,61,62,63,64]. Nevertheless, TDR with the preservation of the azygos vein has been reported during robot-assisted thoracoscopic esophagectomy with no substantial effect on the total number of harvested lymph nodes [65,66,67]. Despite the higher number of retrieved lymph nodes, the real oncologic effect of TDR remains unsolved with some authors reporting even worse 5-year survival rates and more distant organ metastases [56]. Hence, because the lack of robust evidence for short- and long-term outcomes, prospective studies are warranted to assess the clinical and prognostic impact of the preservation of both the thoracic duct and the azygos vein.

Some concerns should be pondered while inferring our results. First, all but one of the included studies described prophylactic TDL; only Fei et al. [24] reported TDL in the case of intraoperative chylous leakage or suspected thoracic duct injury. Notably, we cannot exclude that intraoperative TDL was accomplished in some patients because of inadvertent iatrogenic injury. Hence, this would introduce selection/allocation bias that should be considered while interpreting our data. Second, all included studies were of a retrospective design while allocation bias may have affected our results. However, three studies performed a 1:1 propensity-score matching analysis adjusted for clinically relevant confounders (i.e., age, pathological tumor stage, etc.). The non-adjusted studies by Hou et al. [22] and Yang et al. [26] were well-balanced, whereas no significant differences were detected in the univariate demographic analysis among populations. Third, neoadjuvant and adjuvant therapies changed over the study period and were completed in a limited number of patients; therefore, this potential additional bias should be measured. Fourth, all studies were from tertiary level Eastern centers, and, therefore, the results might not be generalizable considering both the operating surgeon proficiency and genomic/biological tumor patterns.

The principal strength of the present IPD analysis is the appraisal of long-term OS using the RMSTD methodology. RMSTD is gaining increasing consensus in clinical oncology as it is a robust and interpretable tool for assessing the survival benefit, thus allowing, in the present analysis, the estimation of the TDL effect during follow-up [68,69]. It matches the area under the Kaplan–Meier survival curves and is easier to understand compared to RR and HR, which may be misinterpreted because both suppose a constant risk during follow-up [70]. We acknowledge that our study does have some limitations related to the baseline heterogeneity (i.e., patient demographics, comorbidities, etc.), and not the uniform report of oncologic data (i.e., histology, grading, adjuvant treatments compliance, and heterogeneous multidisciplinary perioperative care teams or enhanced recovery after surgery programs). The different surgical approaches, operating surgeon expertise/learning curve, and postoperative complications should be also considered because of the possible effect on long-term survival. Finally, our results may not be generalizable because all included studies were from Eastern countries with the majority of patients being diagnosed with squamous cell carcinoma.

## 5. Conclusions

This study seems to suggest a clinically unfavorable impact of TDL on long-term OS after esophagectomy. Patients undergoing TDL have a significantly higher mortality risk within the first 28 months after the operation compared to patients with noTDL, and this may suggest the need of a rigorous follow-up in these patients.

## Figures and Tables

**Table 1:** Demographic, clinical, and operative data for patients undergoing thoracic duct ligation (TDL) and no thoracic duct ligation (NTDL). Pathologic tumor stage is reported according to the to the 7th and 8th edition of the American Joint Committee on Cancer (AJCC). UT: upper thoracic, MT: middle thoracic, LT: lower thoracic, EGJ: esophagogastric junction. Adk: adenocarcinoma, SCC: squamous cell carcinoma; S-W: Sweet esophagectomy; IL-E: Ivor–Lewis esophagectomy; McK-E: McKeown esophagectomy; MIE: minimally invasive esophagectomy. yrs: years; M: male; F: female. Ret: retrospective design. PSM: propensity score matching. Data are reported as numbers, mean ± standard deviation, and median (range). Nr: not reported.

Author, Year, Contry, Study Design	Study Period	Treatment	No. pts	Age (yrs)	M/F	UT	MT	LT	EGJ	AJCC	Tis-T1	T2	T3	T4	N0	N1	N2	N3	Adk/SCC/Other	S-E	IL-E	McK-E	MIE
Hou et al., 2014, China, Ret [22]	1996–2008	TDL	989	57.2 ± 9.2	786/203	195	591	176	4	7th	61	234	657	30	504	264	166	55	17/930/42	676	22	262	nr
		noTDL	815	58.5 ± 9.8	608/207	126	332	176	179		68	196	528	16	385	217	146	67	170/604/41	576	21	162	nr
Bao et al., 2020, China, Ret PSM [23]	2009–2018	TDL	134	59.5 ± 6.3	105/29	15	104	15	0	nr	9	15	92	13	88	42	4	0	0/134/0	0	0	134	134
		noTDL	40	58.7 ± 7.3	29/11	3	34	3	0		2	4	29	3	29	10	1	0	0/40/0	0	0	40	40
Fei et al., 2020, China, Ret PSM [24]	2012–2014	TDL	185	61.4 ± 7.3	147/38	13	113	57	2	8th	46	49	83	7	100	61	20	4	4/166/15	104	31	50	39
		noTDL	185	61.1 ± 7.9	139/46	11	125	48	1		49	50	81	5	98	57	20	10	4/165/16	97	29	59	45
Chen et al., 2020, China, Ret PSM [25]	2003–2013	noTDL	437	58.7 ± 8.2	342/95	90	315	32	0	7th	54	85	290	8	196	126	90	25	nr	0	104	333	nr
		noTDL	437	59.1 ± 7.8	335/102	88	305	44	0		68	90	267	12	209	139	66	23	nr	0	110	327	nr
Yang et al., 2022, China, Ret [26]	2016–2021	TDL	33	>60:19	28/5	5	18	10	0	8th	17	10	6	0	nr	nr	nr	nr	nr	nr	nr	nr	69
		noTDL	36	>60:18	29/7	6	16	14	0		16	12	8	0	nr	nr	nr	nr	nr	nr	nr	nr	

**Table 2:** Overall survival. The 60-month restricted mean survival time difference (RSMTD) at different time horizons for noTDL vs. TDL comparison. SE: standard error; 95% CI: confidence intervals; mos: months.

Time Horizon	No. Trials	RMSTD (mos)	SE	95% CI	*p* Value
12-month	5	−0.2	0.11	−0.43, −0.01	0.049
24-month	5	−1.1	0.4	−1.8, −0.4	0.005
36-months	5	−2.1	0.6	−3.3, −0.7	0.003
48-months	5	−2.8	0.9	−4.8, −0.8	0.004
60-months	5	−3.5	1.2	−6.1, −0.8	0.009

**Table 3:** Overall survival. Time-dependent hazard ratio (HR) analysis for the TDL vs. noTDL. Table legend. 95%CI: confidence interval; mos: months.

	TDL vs. NTDL HR (95% CI)
6 mos	1.83 (1.53–2.21)
12 mos	1.54 (1.38–1.73)
24 mos	1.21 (1.12–1.35)
28 mos	1.14 (1.02–1.28)
36 mos	1.04 (0.90–1.19)
48 mos	0.95 (0.82–1.12)
60 mos	0.96 (0.79–1.17)

## Data Availability

The data collected and analyzed during the current review are available from the corresponding author upon reasonable request.

## Supplementary Materials

The following supporting information can be downloaded at: https://www.mdpi.com/article/10.3390/jcm13102849/s1, Table S1: Quality assessment of the included studies (ROBINS-I tool). Table S2: Different techniques for TDL according to the included studies. Table S3: Definition of postoperative chylothorax according to the included studies. Table S4: The certainty of evidence assessed with the GRADE methodology.
