# Supplementary material for: Does Thoracic Duct Ligation at the Time of Esophagectomy Impact Long-Term Survival? An Individual Patient Data Meta-Analysis
Source: J Clin Med. 2024 May 12;13(10):2849. doi: 10.3390/jcm13102849 (PMC11122204; doi:10.3390/jcm13102849)
Supplement: Supplementary file 1 [file jcm-13-02849-s001.zip › Suppl Table 1 ROBBINS-I.pdf]

Study	Confounding Bias	Selection Bias	Classification Bias	Intervention Bias	Missing Data Bias	Measurement Bias	Reporting Bias	Bias
Hou et al., 2014	Serious	Moderate	Low	Low	Low	Moderate	Moderate	Moderate
Bao et al., 2020	Moderate	Low	Low	Low	Low	Moderate	Moderate	Moderate
Fei et al., 2020	Moderate	Low	Moderate	Low	Moderate	Moderate	Moderate	Moderate
Chen et al., 2020	Moderate	Low	Moderate	Low	Low	Low	Moderate	Moderate
Yang et al., 2022	Moderate	Serious	Moderate	Moderate	Moderate	Moderate	Moderate	Serious

Supplementary Table 1. Quality assessment of the included studies (ROBINS-I tool). Each domain is evaluated with one of the following: Low, Moderate, Serious, and Critical. The categories of judgement for each study are low, moderate, serious, and critical risk of bias.
