# Supplementary material for: Does Thoracic Duct Ligation at the Time of Esophagectomy Impact Long-Term Survival? An Individual Patient Data Meta-Analysis
Source: J Clin Med. 2024 May 12;13(10):2849. doi: 10.3390/jcm13102849 (PMC11122204; doi:10.3390/jcm13102849)
Supplement: Supplementary file 1 [file jcm-13-02849-s001.zip › Suppl Table 2.pdf]

**Author, year, contry**	**Tecnhique for TDL**
Hou et al., 2014	Mass ligation
Bao et al., 2020	Mass ligation<br>(5cm above the diaphragm)
Fei et al., 2020	Mass ligation<br>(2cm above the diaphragm)
Chen et al., 2020	Mass ligation<br>(8th – 9th thoracic vertebra)
Yang et al., 2022	Mass ligation<br>(2cm above the diaphragm)

Supplementary Table 2. Different techniques for TDL according to the included studies.
