# Supplementary material for: Does Thoracic Duct Ligation at the Time of Esophagectomy Impact Long-Term Survival? An Individual Patient Data Meta-Analysis
Source: J Clin Med. 2024 May 12;13(10):2849. doi: 10.3390/jcm13102849 (PMC11122204; doi:10.3390/jcm13102849)
Supplement: Supplementary file 1 [file jcm-13-02849-s001.zip › Suppl Table 3.pdf]

Author, year	Definition of Chylothorax
Hou et al., 2014	Postoperative chylothorax refers to the postoperative chylothorax that underwent surgical intervention
Bao et al., 2019	Diagnosis was made by biochemical analysis of fluid. Laboratory analysis revealed high triglyceride levels in the fluid (> 1.24 mmol/L).
Fei et al., 2020	Postoperative chylothorax was defined as triglyceride levels>110 mL/day or by the presence of chylomicrons found in pleural fluid.
Chen et al, 2020	Then the Lipoprotein staining for chylomicrons would be considered to confirm the diagnosis. Otherwise, the chest fluid biochemical analysis with triglyceride levels greater than 1.24mmol/L (110mg/dL) or cholesterol less than 5.18mmol/L (200mg/dL) were also identified as chylothorax.
Yang et al., 2022	nr

Supplementary Table 3. Definition of postoperative chylothorax according to the included studies.
