# Supplementary material for: Does Thoracic Duct Ligation at the Time of Esophagectomy Impact Long-Term Survival? An Individual Patient Data Meta-Analysis
Source: J Clin Med. 2024 May 12;13(10):2849. doi: 10.3390/jcm13102849 (PMC11122204; doi:10.3390/jcm13102849)
Supplement: Supplementary file 1 [file jcm-13-02849-s001.zip › Suppl Table 4.pdf]

5-year Overall Survival

Certainty assessment							№ of patients		Effect		Certainty	Importance
№ of studies	Study design	Risk of bias	Inconsistency	Indirectness	Imprecision	Other considerations	[intervention]	[comparison]	Relative (95% CI)	Absolute (95% CI)		
5	non-randomised studies	not serious	not serious	serious	serious	strong association			-	SMD 3.5 SD lower (6.1 lower to 0.8 lower)	⨁⨁⨁◯ Moderate	IMPORTANT

CI: confidence interval; SMD: standardised mean difference

Postoperative chylothorax

Certainty assessment							№ of patients		Effect		Certainty	Importance
№ of studies	Study design	Risk of bias	Inconsistency	Indirectness	Imprecision	Other considerations	[intervention]	[comparison]	Relative (95% CI)	Absolute (95% CI)		
5	non-randomised studies	not serious	not serious	not serious	not serious	all plausible residual confounding would reduce the demonstrated effect	22/1814 (1.2%)	23/1538 (1.5%)	RR 0.66 (0.28 to 1.56)	5 fewer per 1.000 (from 11 fewer to 8 more)	⨁⨁⨁⨁ High	IMPORTANT

CI: confidence interval; RR: risk ratio

Supplementary Table 4. The certainty of evidence assessed with the GRADE methodology.
